# Effectiveness and Safety of Robotic Radiosurgery for Optic Nerve Sheath Meningiomas: A Single Institution Series

**DOI:** 10.3390/cancers13092165

**Published:** 2021-04-30

**Authors:** Carolin Senger, Anne Kluge, Melina Kord, Zoe Zimmermann, Alfredo Conti, Markus Kufeld, Anita Kreimeier, Franziska Loebel, Carmen Stromberger, Volker Budach, Peter Vajkoczy, Gueliz Acker

**Affiliations:** 1 Department of Radiation Oncology, Charité Universitätsmedizin Berlin, Corporate Member of Freie Universität Berlin, Humboldt-Universität zu Berlin, and Berlin Institute of Health, Augustenburger Platz 1, 13353 Berlin, Germany; anne.kluge@charite.de (A.K.); melina.kord@charite.de (M.K.); anita.kreimer@charite.de (A.K.); carmen.stromberger@charite.de (C.S.); volker.budach@charite.de (V.B.); 2 Charité Cyberknife Center, Augustenburger Platz 1, 13353 Berlin, Germany; zoe.zimmermann@charite.de (Z.Z.); alfredo.conti2@unibo.it (A.C.); markus.kufeld@cyber-knife.net (M.K.); franziska.loebel@charite.de (F.L.); peter.vajkoczy@charite.de (P.V.); gueliz.acker@charite.de (G.A.); 3 Department of Neurosurgery, Charité Universitätsmedizin Berlin, Corporate Member of Freie Universität Berlin, Humboldt-Universität zu Berlin, and Berlin Institute of Health, Charitéplatz 1, 10117 Berlin, Germany; 4 Alma Mater Studiorum-Università di Bologna, Dipartimento di Scienze Biomediche e Neuromotorie (DIBINEM), Via Altura 3, 40139 Bologna (BO), Italy; 5 IRCCS Istituto delle Scienze Neurologiche di Bologna, Via Altura 3, 40139 Bologna (BO), Italy; 6 Berlin Institute of Health at Charité Universitätsmedizin Berlin, BIH Acadamy, Clinician Scientist Program, Charitéplatz 1, 10117 Berlin, Germany

**Keywords:** Cyberknife, robotic radiosurgery, stereotactic radiosurgery, optic nerve sheath meningioma, hypofractionation, multisession

## Abstract

**Simple Summary:**

Optic nerve sheath meningiomas (ONSM) are a rare subtype of meningioma. Only four retrospective studies with 3–21 patients have been published on the treatment of ONSM by radiosurgery. This study represents the largest published series on robotic radiosurgery to date, treating 25 patients with 27 ONSM lesions. Furthermore, hypofractionated radiosurgical treatment proves to be a safe alternative to surgery and fractionated stereotactic radiation with an overall local tumor control rate of 96.0% and stable or improved visual acuity in 90.0% and 10.0% of patients, respectively. We believe that our study makes a significant contribution to the literature, as our results indicate that robotic radiosurgery is a safe and effective treatment for the management of ONSM and offers a potential treatment option that would improve patient care and clinical outcomes.

**Abstract:**

The role of robotic radiosurgery (RRS) in the treatment of optic nerve sheath meningiomas (ONSM) remains controversial and it is only performed in specialized institutions due to tight dose constraints. We evaluated the effectiveness and safety of RRS in the management of ONSM. Twenty-five patients with 27 ONSM lesions who underwent RRS using the Cyberknife (CK) system were retrospectively analyzed (median age, 47.9 years; 84.0% women). Multisession RRS was used with 4–5 fractions with a cumulative dose of 20.0–25.0 Gy in 84.0% of patients and a single fraction at a dose of 14.0–15.0 Gy in 16% of patients. Prior to RRS, seven (28%) patients experienced blindness on the lesion side. In those patients with preserved vision prior to radiosurgery, the visual acuity remained the same in 90.0% and improved in 10.0% of the patients. Overall local tumor control was 96.0% (mean follow-up period; 37.4 ± 27.2 months). Neither patient age, previous surgery, or the period from the initial diagnosis to RRS showed a dependency on visual acuity before or after radiosurgery. RRS is a safe and effective treatment for the management of ONSM. Hypofractionation of radiosurgery in patients with preserved vision before CK treatment results in stable or improved vision.

## 1. Introduction

Optic nerve sheath meningiomas (ONSM) are tumors of the anterior visual pathway, occurring at a rate of approximately 1–2% of all intracranial meningiomas, predominantly affecting middle-aged females [1,2]. The most frequent symptoms at the initial diagnosis are impaired visual acuity up to complete loss of vision, visual field or color vision defects, and exophthalmos [2]. Of pathognomonic significance for ONSM is the triad of gradual painless visual loss, atrophy of the optic nerve, and optociliary shunt vessels [3,4]. ONSM are classified as primary or secondary (perioptic), depending on the origin, with the primary type occurring less frequently (1:9) [5,6]. Secondary ONSM extend from intracranial structures into the orbit, located < 2–3 mm away from the optical structures [7]. In cases of progression, primary ONSM may spread intracranially within the optic nerve canal and involve the optic chiasm causing bilateral vision defects due to direct extension or by tension and straining of the optic chiasm. The presence of bilateral tumors occurs in about 5–10% of ONSM patients, caused by overgrowth from the opposite side. However, some are independent tumors, occasionally associated with neurofibromatosis type 2 [1,2,8,9].

The treatment of these lesions is challenging because of their intimidate relationship with the optic nerve. Traditional treatment strategies for ONSM are observation, which can lead to progressive visual impairment and exophthalmos, microsurgical resection, and/or radiation therapy. So far, none of these methods has clearly emerged as the treatment of choice for ONSM [10]. Surgery is usually reserved for patients with severe or complete visual loss or with relevant exophthalmos. The circumferential envelopment of the nerve by the meningioma makes it impossible to perform a complete resection in most patients while avoiding serious damage to the optic nerve or vasculature [11]. The substantial risk of visual loss after microsurgery can be mitigated by a non-surgical treatment approach. In this regard, conventional radiation therapy has an important role in primary or adjuvant irradiation of ONSM [12]. Currently, fractionated stereotactic radiotherapy (FSRT) is increasingly used and has proven to be effective and reasonably tolerable, with a cumulative dose of 50.4–54.0 Gy [1,13,14,15,16,17]. In the majority of treated patients, FSRT can preserve visual function, with comparatively few risks for side effects [1,14].

The value of robotic radiosurgery (RRS) for the treatment of ONSM is still a topic of debate. Compared to FSRT, RRS achieves extremely narrow dose distribution and improved dose conformality while focusing the beams on the meningioma that maximizes the preservation of the organ at risk [18]. Gammaknife-based radiosurgery treatments were rarely used in ONSM because the fixed frame required the dose to be applied in a single fraction, thus exceeding the dose tolerance of the visual pathway [15]. Cyberknife (CK) offers submillimeter accuracy despite frameless irradiation, allowing the dose to be divided into multiple fractions, referred to as hypofractionated or multisession RRS [16,19,20].

Until now, RRS for ONSM has only been performed in a few specialized institutions because of the substantial technical requirements and tight dose constraints of the optic pathway. The purpose of this research was to evaluate the clinical outcomes of CK-RRS in the management of our ONSM patient cohort in order to assess the feasibility of RRS as a valuable treatment option.

## 2. Materials and Methods

### 2.1. Study Design

This retrospective study of clinical and technical data from our patients was authorized by the Charité ethics committee (EA1/036/20). All study participants provided informed consent. We included all patients with ONSM who received RRS at the Charité Cyberknife Center, Berlin, Germany, over a 7-year period between October 2012 and November 2019.

### 2.2. Variables

Data on patient clinical characteristics, previous surgical treatments, and vision prior to radiosurgery were evaluated. We also analyzed the growth pattern, localization, and configuration (circularly = tubular, globular, fusiform, or peripherally = focal enlargement of the optic nerve) based on a previous description from the literature [21]. We evaluated clinical outcomes with regard to vision preservation and local tumor control (LC) on the basis of post-treatment imaging and safety. The visual acuity in decimal before and after CK-RRS was categorized according to the World Health Organization (WHO) classification in category 0—mild or no visual impairment (visual acuity equal to or better than 0.3), category 1—moderate visual impairment (visual acuity equal to or better than 0.1 but worse than 0.3); category 2—severe visual impairment (visual acuity equal to or better than 0.05 but worse than 0.1), category 3—profound visual impairment/count fingers at 1 m (visual acuity equal to or better than 0.02 but worse than 0.05), category 4—near blindness/light perception (visual acuity equal worse than 0.02), and category 5—blindness/no light perception [22,23].

Movement of the optic nerve and the influence of the dose calculation algorithm on the dose distribution for the ipsilateral optic nerve were further analyzed. Dose differences per fraction for the ipsilateral optic nerve near the maximum dose due to treatment uncertainties were calculated. We further analyzed the dose-volume parameters, which included treatment dose, fractionation concept, treated volume, mean, and maximum doses for the target and organs at risk (OAR).

### 2.3. Cyberknife Treatment

CK-RRS treatment was recommended at the Charité Neurooncology Tumor Conference. For immobilization, a thermoplastic mask was manufactured for each patient individually. Contrast-enhanced thin-slice planning computed tomography (CT) (0.75 mm) and coregistered magnetic resonance imaging (MRI) datasets (contrast-enhanced T1-weighted fast three-dimensional gradient-echo and T1-weighted fat-suppressed images) served as a basis for treatment planning. The expansion of the ONSM based on CT and MRI datasets was contoured, adding 0–1 mm safety margin for generating the planning target volume (PTV). The involved optic nerve was contoured in the coronal planes, strictly avoiding overlap with the PTV in patients with residual vision. In general, hypofractionated RRS was used for all patients with visual acuity WHO category 1–4. Single-fraction RRS was only applied in patients with complete vision loss of the treated side (visual acuity WHO category 5).

The RRS treatment planning and dose calculations were performed using MultiPlan 4.5/Precision 2.0, while the dose was applied using the Cyberknife Radiosurgery System VSI (Accuray Inc., Sunnyvale, CA, USA). The Ray-Tracing algorithm for dose calculation was used routinely. The linear-quadratic model, with an a/ß ratio of 3 Gy for ONSM equivalent dose in 2 Gy per fraction (EQD2_3_), and 2 Gy for OAR (EQD2_2_) was applied to calculate radiation doses in equivalent 2 Gy fractions. Critical OAR to be preserved are the ipsilateral and contralateral optic nerves, retina/eye, chiasma, and brainstem. The following dose constraints to the optic pathway for five fractions of RRS were considered: <0.20 cm³ of the visual pathway was allowed to receive 23.0 Gy with a maximum dose of 25.0 Gy in ≤0.035 cm^3^ [24]. Adjustments were made to the treatment dose, fractionation, or isodose when the OAR dose was exceeded based on the individual meningioma extension. To prevent edema-related headache and transient visual disturbances, patients received 4 mg of dexamethasone as needed after each fraction of RRS.

### 2.4. Follow-Up

The treatment response was assessed by post-radiosurgery MRI at 6 months and then annually. In this analysis, we included the most recent follow-up. The focus regarding the patient’s clinical outcomes was visual acuity. In addition, we performed follow-up calls to all patients to inquire about subjective visual responses. Response to treatment of each ONSM was assessed by current MRI extent compared with the former PTV for local control: (1) stable disease (SD), defined as no change in the size of the tumor; (2) partial remission (PR), defined as any tumor volume reduction, and (3) progressive disease (PD), defined as any tumor growth in at least 2 consecutive MRI scans. Progression-free survival (PFS) was calculated from the end of CK-RRS until the last available follow-up or PD. Visual acuity at baseline and last available follow-up were evaluated according to the WHO classification.

### 2.5. Analysis of the Dose Distribution

For dose evaluations, both optic nerves of all patients were contoured in the planning CT and in the coregistered MRI sequence. Maximal (95th percentile of the Hausdorff distance) and average symmetric surface distances between the optic structures of MRI and CT were determined for each patient. Overlays with optic chiasm or PTV were not allowed. Volume deviations were calculated to provide information about optic nerve mobility, to estimate necessary safety margins, and to take into account possible dose variations for the PTV and the optic nerve for subsequent patients. Dose distributions were recalculated with the Monte Carlo algorithm (Gaussian smoothing: 1.0, uncertainty: 1.0%) of precision 2.0 (Accuray Inc., Sunnyvale, CA, USA) as it is more accurate in such heterogeneous tissue of the orbital cavity with bone, air, and soft tissue transitions. All digital imaging and communications (DICOM) radiotherapy structures and both dose distributions (Monte Carlo and Ray-Tracing) were exported, and motion pattern evaluation and dosimetric comparisons were performed with Python 3.6 using the packages NumPy, Pydicom, Skimage, and MedPy.

### 2.6. Statistics

Descriptive parameters were reported as mean ± standard deviation, median, and range. Statistical analyses were performed using the Statistical Package for the Social Sciences software (version 25, IBM, Armonk, NY, USA). Due to the small number of patients included in this study, non-parametric data distribution was assumed. To compare visual acuity before and after CK-SRS in the same subjects, the Wilcoxon signed-rank test was used. The Mann–Whitney-U test or Kruskal–Wallis test was applied for group comparisons. Results were declared as significant for *p*-values of <0.05. Mean PFS was estimated using the Kaplan–Meier analysis.

### 2.7. Research of the Literature

**Figure 1:**
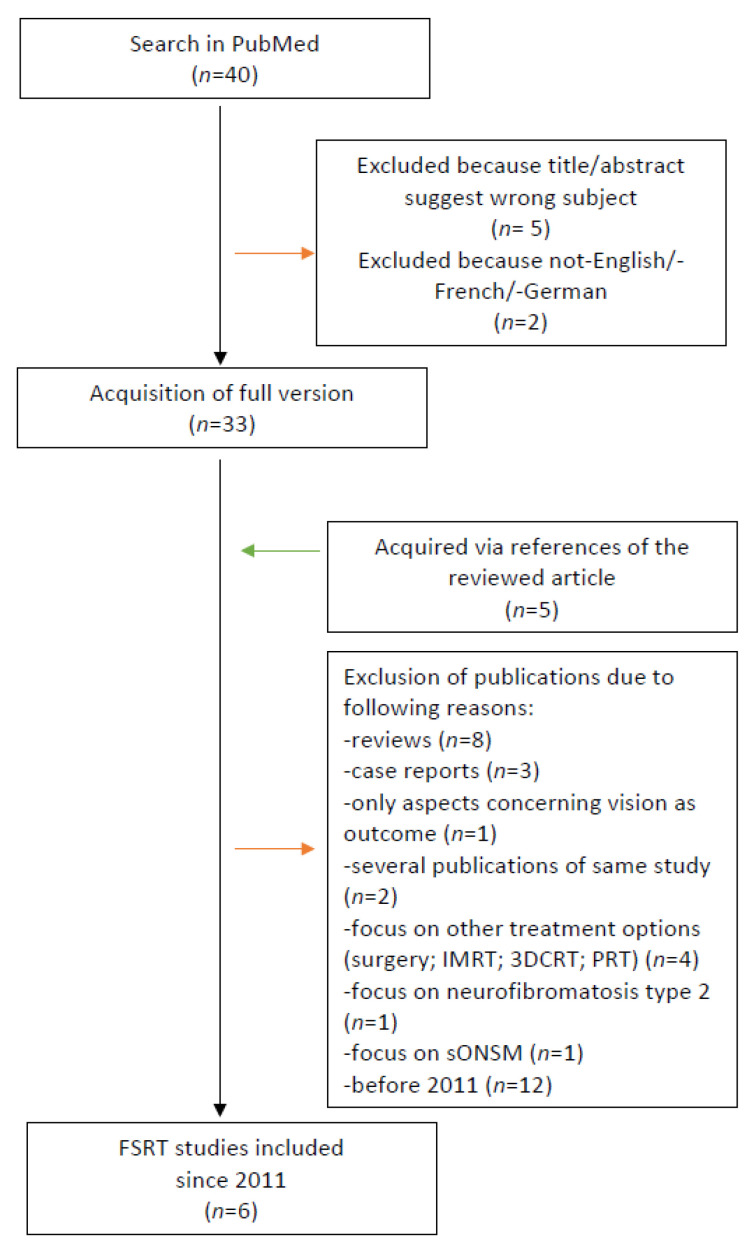
Selection process of studies for fractionated stereotactic radiotherapy (FSRT).

The local control rate and side effects of radiosurgery treatments and FSRT of ONSM were reviewed. The search was performed using the PubMed database between the 6th and 8th May 2020 and included the following free text terms: “Fractionated stereotactic radiotherapy optic nerve sheath meningioma”, “FSRT optic nerve sheath meningioma”, “stereotactic radiotherapy (SRT) optic nerve sheath meningioma” for FSRT and “stereotactic radiosurgery (SRS) optic nerve sheath meningioma”, “Cyberknife optic nerve sheath meningioma”, and “Gammaknife optic nerve sheath meningioma” for radiosurgery. Publications in English, French, or German and studies focusing on secondary ONSM, neurofibromatosis, or other treatments were excluded. The remaining studies were acquired in full version and their eligibility was assessed. The references of the included studies were manually checked for further relevant publications. Figure 1 demonstrates the search process for FSRT. The study characteristics, methods, and outcomes were collected in tables.

## 3. Results

### 3.1. Patient Characteristics

A total of 25 patients with 27 ONSM lesions were identified, and two patients (8.0%) had bilateral tumors. The majority of lesions were on the right side (63.0%). The median age was 47.9 years (mean age: 48.2 ± 15.2 years) with a range of 11.4–75.5 years. The female-to-male ratio for ONSM was 3:1 (Table 1). The median time interval from meningioma diagnosis to CK-SRS was 5.4 (range: 1.0–139.7) months. Of the 27 ONSM lesions, the majority (92.6%) presented a circular growth pattern, while only 7.4% presented a peripheral growth pattern as a sign of an early tumor stage. The involvement of the optic canal was both orbital and canalicular in half of the cases (51.9%). A pre-SRS visual function assessment was available for all patients. Visual acuity was reduced in all patients and below 0.6 in 21 out of 27 patients with ONSM (77.8%). A total of 10 lesions (37.0%) were classified as WHO category 0 with a mild reduction of visual acuity followed by 7 lesions (25.9%) with the most impairment as category 5. Two patients with bilateral ONSM were blind in one eye. Of the patients with ONSM with category 4–5 visual acuity prior to CK-SRS, 54.6% initially underwent surgical decompression with volume reduction. Of the remaining 16 out of 27 patients with a visual acuity WHO grade 1–3, 68.8% had additional visual field deficits. In Table 2 we present a comparison of the patient cohorts with and without a previous surgery. Other frequent clinical signs and symptoms according to the pre-SRS evaluation were exophthalmos and decreased motility in 29.6% of patients with ONSM. A total of nine patients (36.0%; 33.3% of the lesions) were operated previously with optic canal decompression. Of those who underwent previous surgery, all had a histologically proven diagnosis of WHO grade 1 meningioma. For all others, no biopsies were obtained for histological confirmation. Ten patients received a 68Ga-DOTATOC-PET/MRI before CK-SRS to support the clinical and MRI-based diagnosis of meningioma. Thus, a total of 15 patients had a confirmed diagnosis of ONSM (three patients received both PET and histologic confirmation). An overview of the pretreatment patient and lesion characteristics, including tumor growth patterns and ophthalmological features, is reported in Table 1.

Visual acuity groups before radiosurgery showed no difference in patient age, lesion volume, or time from initial diagnosis to CK-SRS (*p* = 0.470; *p* = 0.115; *p* = 0.768; Kruskal–Wallis test). The previously operated cohort showed significantly worse visual acuity before CK-SRS (mean WHO for previously operated 3.6; not operated 1.6; *p* = 0.042; Fisher’s exact test), probably due to the fact that more than half of the patients had already only WHO 4–5 visual acuity prior to surgery.

### 3.2. Treatment Characteristics

**Figure 2:**
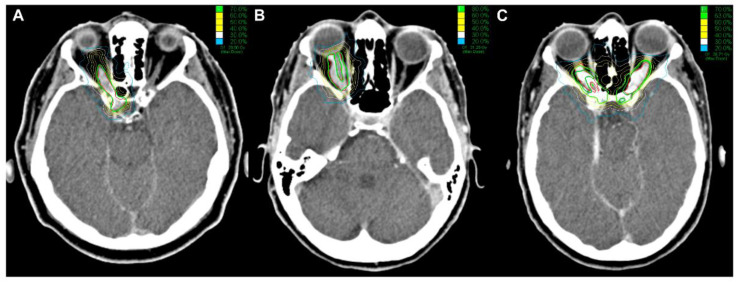
Cyberknife treatment planning showing axial planning-CT of a: (**A**) right sided ONSM without vision treated with 1 × 14 Gy (70% isodose); (**B**) right sided ONSM treated with 5 × 5 Gy (80% isodose) for vision preservation; (**C**) bilateral ONSM treated with 5 × 5 Gy (70% isodose) for the left and 5 × 4.5 Gy for the right side (red lines: PTV, green lines: prescription isodose).

Patients were treated in five (*n* = 11), four (*n* = 12), and one fraction (*n* = 4). Individual fractions of the CK-SRS were applied at intervals of 24 h. Multisession SRS with 4–5 fractions and a cumulative dose of 20.0–25.0 Gy was used in 84.0% of patients, while the remaining 16.0% of patients received single-fraction SRS at a dose of 14.0–15.0 Gy. All patients who received single-fraction SRS were already blind prior to irradiation (Table 2). Treatment doses were prescribed to a mean isodose line of 76.0%, ranging from 70.0% to 85.0%. Radiosurgery was delivered to a mean PTV of 1.80 ± 2.62 cm^3^ (median 0.96 cm^3^, range 0.12–14.10 cm^3^, Table 2). Irradiation planning examples for single-fraction SRS, hypofractionated SRS, and CK-SRS of the bilateral ONSM are shown in Figure 2.

Overall, the mean EQD2_3_ of the PTV was 46.8 Gy with a median of 43.5 Gy (range: 36.1–80.4) and 41.7 Gy with a median of 39.7 (range: 36.1–53.0 Gy) for patients with visual acuity < 5 according to WHO category.

For patients with residual vision (WHO category < 5), maximal EQD2_2_ doses to the ipsilateral and contralateral optic nerve and chiasma were 43.8 ± 9.8 (median 43.1 Gy, range 26.5–62.0 Gy), 2.0 ± 1.5 (median 1.4 Gy, range 0.5–5.7 Gy), and 5.9 ± 9.3 (median 1.6 Gy, range 0.1–28.8 Gy), respectively. Table 3 shows an overview of dose-volume parameters for the PTV (near minimum dose (D_min_), mean dose (D_mean_), and near maximum dose (D_max_)), ipsilateral optic nerve (D_max_ and D_mean_), optic chiasm (D_max_), contralateral optic nerve (D_max_), ipsilateral retina/eye (D_max_), and ipsilateral lens (D_max_) of all treatment plans.

### 3.3. Local Tumor Control

**Figure 3:**
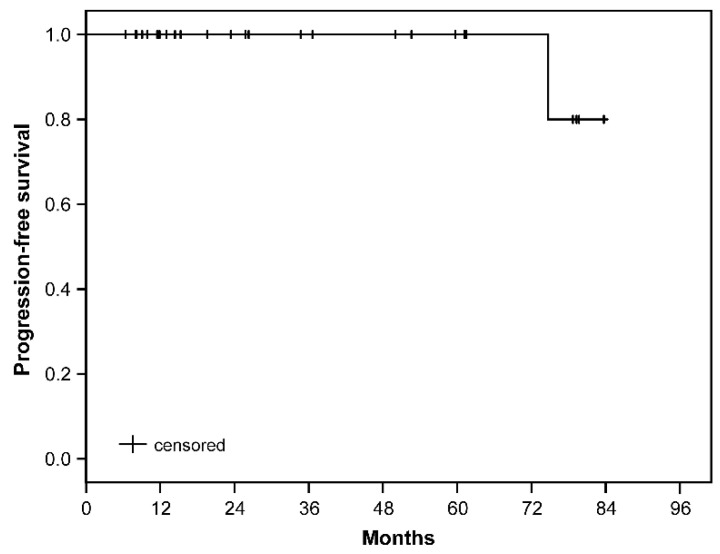
Kaplan–Meier curve of the progression-free survival. Patients at risk after 6-, 12-, 24-, 36-, and 72 months after SRS were 27, 19, 13, 11, and 4, respectively.

Follow-up MRI and visual acuity tests were available for all cases. The overall LC was 96.0%, with a mean follow-up time of 37.4 ± 27.2 months and a range of 6.4–83.7 months. In particular, 85.2% of the lesions showed an SD (no change), a PR was achieved in 11.1%, while only one lesion (4.0%) suffered from a PD. The mean PFS was 80.7 months (confidence interval: 75.9–85.5). The estimated local tumor PFS rates at 6, 12, 24, 36, and 72 months after SRS were 100%, 100%, 100%, 100%, and 80.0%, respectively (Figure 3).

The only patient in whom tumor progress was detected was a 30.9-year-old man with a history of two surgeries and blindness prior to CK-SRS. Tumor progression occurred within the treatment field. The patterns of failure analysis showed that the patient also had the largest PTV (14.1 cm^3^). The patient underwent microsurgical resection of the tumor progress 70.9 months after CK-SRS. Histology confirmed the diagnosis of WHO grade 1 meningioma due to the low proliferation index (2 mitosis/10 high power field, proliferation rate 3%) and the presence of only two atypical criteria (patternless growth, necrosis).

### 3.4. Morbidity and Outcome

**Figure 4:**
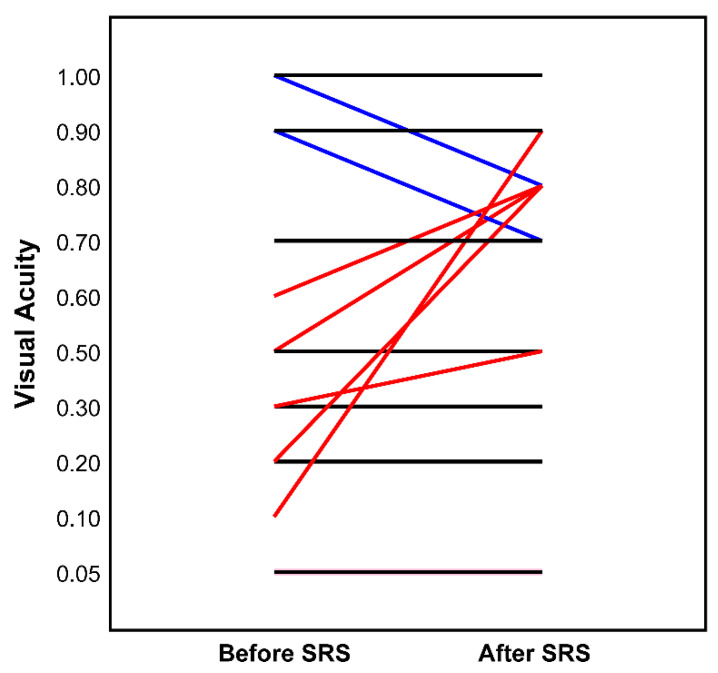
Visual acuity (decimal) before and after (latest available follow-up) Cyberknife (*n* = 16, excluded: WHO > 4). Each line represents one patient: black—idem, red—improved, blue—impaired visual acuity.

Apart from a mild headache (*n* = 1) and complaint of transient diplopia (*n* = 1), which both responded to short-term dexamethasone 4 mg, no other acute morbidity was observed. Visual acuity, except in patients with initial blindness on the treated side, remained unchanged in 90.0% (18 lesions), improved in 10.0% (2 lesions), and did not deteriorate in any patient according to the WHO classification of severity of visual impairment. For patients with a measurable visual acuity (*n* = 16, excluded: WHO > 4), vision test results before and after CK-RRS are shown in Figure 4. No significant difference was found between visual acuity before and after irradiation (Wilcoxon signed-rank test, *p* = 0.121). Neither the dose to the ipsilateral optic nerve, nor the treated volume, nor the period from the initial diagnosis to CK-RRS had an impact on the change in visual acuity (*p* = 0.864; *p* = 0.827; *p* = 0.353; Kruskal–Wallis test). No other adverse effects were observed.

### 3.5. Optic Nerve Movement and Dose Uncertainties

**Figure 5:**
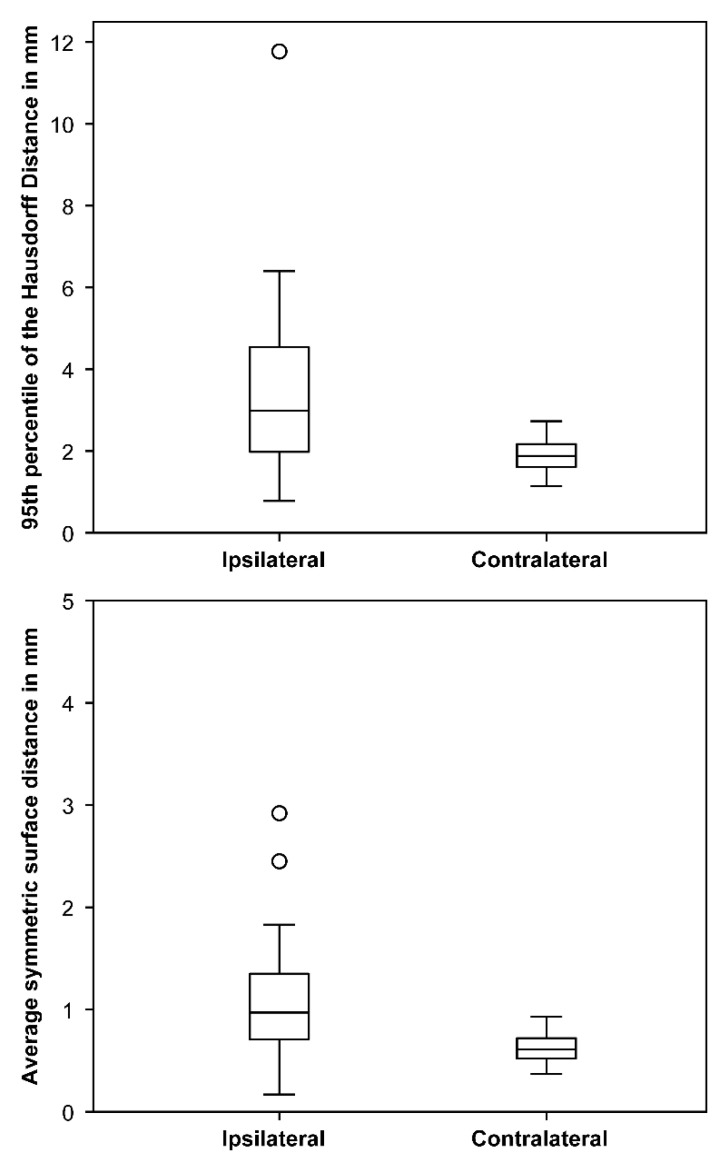
Maximal (95th percentile of the Hausdorff distance) and average symmetric surface distances between optic nerves contoured in MRI and CT in mm indicating larger differences for the ipsilateral nerve. Boxplots showing median and interquartile range, whiskers indicate maximum and minimum within a 1.5 interquartile range, circles indicate outliers.

The motion of the ipsilateral and contralateral optic nerves estimated from the CT and MRI contours were significantly different. The median maximal deviations between contours of MRI and CT were 2.99 mm (range: 0.78–11.77 mm) for the ipsilateral optic nerve and 1.88 mm (range: 1.14–2.73 mm) for the contralateral optic nerve (Mann–Whitney U test; *p* < 0.001). The median average deviations between contours of MRI and CT were 0.97 mm (range: 0.17–2.92) for the ipsilateral optic nerve and 0.61 mm (range: 0.37–0.93) for the contralateral optic nerve (Mann–Whitney U test; *p* < 0.01, Figure 5). Table 2 demonstrates the estimated mobility of the ipsilateral optic nerve in different patient cohorts.

This estimated optic mobility leads to an average maximal dose difference in the optic nerves of 0.1 ± 0.4 Gy (range: −0.5–1.1) per fraction for the treated side. The inhomogeneous tissue structure in the orbital region resulted in a dose difference of 0.3 ± 0.2 Gy (range: 0.1–0.7) per fraction for the maximal optic nerve dose. Therefore, the doses of the Monte Carlo algorithm were always higher.

### 3.6. Summary of the Literature

The search for studies of CK or Gammaknife radiosurgery in ONSM resulted in six publications. Four of them were included in this review (Table 4) [18,25,26,27]. The case report by Mokhtarzadeh et al. [28] was excluded as it did not address local control and side effects after treatment. The study by Milano et al., (2018) [29] was also excluded because it investigated the dose tolerance of the optic pathways; it specifically excludes patients with ONSM, thus making it inappropriate for this review.

The search for FRST in ONSM revealed 21 publications. Due to the large number of publications on FSRT, only studies from 2011 onwards were included (Table 5) [1,15,30,31,32,33]. A case report by Inoue et al. [19] and a review and retrospective study by Pacelli et al. [20] were excluded due to the limited number of patients (*n* = 1 and *n* = 5, respectively) compared to the FSRT studies listed.

## 4. Discussion

Our study represents the largest reported ONSM series treated with single or hypofractionated CK-RRS, underscoring the efficacy and safety of this technique for ONSM. Our results highlight that, particularly with the use of hypofractionated RRS, sufficient local control of ONSM can be achieved with remarkably few side effects. In addition, the analysis of the nerve movement and the calculation algorithm revealed only small dose uncertainties for the target and visual pathways.

The origin and location of ONSM make it one of the greatest challenges in radiosurgery, best managed interprofessionally by the ophthalmology, neurosurgery, and radiation oncology departments. The use of RRS for the treatment of ONSM is still limited to a few specialized centers (Table 4). Previous studies with a small number of patients (*n* ≤ 5) on hypofractionated RRS by Romanelli et al. [18,25] showed encouraging results and revealed that CK offered the highest level of conformity and accuracy. Additionally, the flexibility to irradiate multiple fractions improved the tolerance of the optic nerve, enabling the treatment of larger lesions while preserving visual function. The proportion of patients with ONSM at our center (25/282 of meningioma patients, 8.9%) treated with CK is a multiple of previously reported cohorts of 1–2% in the literature [1,2]. The highest number of patients in our treatment center was six patients per year in 2019 compared to two patients in 2012, showing an upward tendency for SRS and/or indicating an increasing incidence. An increased incidence of ONSM has also been demonstrated by a danish retrospective analysis from Lindegaard et al. [34], most likely due to more frequent use of MRIs and their improved technology in the past years. Similar to what is described in the literature with approximately 5–10% bilateral ONSM and 8.0% of our patients had bilateral lesions [1,2]. ONSM is more common in females, according to a bigger review of ONSM published by Dutton et al. [2]. In our study, the proportion of women was 84.0% with a mean age of 48.2 years. In middle-aged women with visual disturbances, multiple sclerosis may also be suspected as a differential diagnosis. MRI, especially gadolinium-enhanced fat-suppressed MRI sequences, represents the gold standard for diagnosis and has replaced the requirement for a biopsy [35]. Occasionally, optic nerve gliomas cannot be clearly distinguished from ONSM by imaging. One diagnostic tool that can be used to verify ONSM diagnosis is 68Ga-DOTATOC-PET imaging [36]. In our study, the most common symptom prior to SRS was impairment of visual acuity. Additionally, 68.8% of the patients with ONSM had visual field deficits and 29.6% of patients with ONSM had exophthalmos or decreased motility. In previous studies, the most common symptoms at initial diagnosis were similar, including impaired visual acuity, visual field or color vision defects, and exophthalmos [2].

The establishment of hypofractionated radiosurgery treatments with up to five fractions, however, allows supposedly lower toxicity to the critical neuronal structures [18,25,27]. At our institution, 84.0% of the patients received a hypofractionated RRS with 4–5 fractions with a total dose of 20.0–25.0 Gy (70.0–85.0% isodose). The achieved LC was 96.0% with a mean follow-up time of 37.4 months. As these tumors generally show slow remission after completion of the CK-RRS due to the benign nature of the disease, SD and PR were achieved in 85.2% and 11.1% of the lesions, respectively. In our cohort, only one patient presented with a progressive disease. The estimated PFS was calculated on this basis. It should be noted that the statistical power of this analysis is limited due to the small number of patients and a single event with an overall heterogeneous disease follow-up time. Even so, the calculated PFS is consistent with the benign nature of the disease. With regard to effectiveness of the CK-RRS, this analysis has to be repeated in the future with longer follow-up periods.

No significant difference was observed between visual acuity before or after irradiation. Neither the dose to the ipsilateral optic nerve, treated volume, or period from the initial diagnosis to CK-SRS had an impact on the change in visual acuity after SRS treatment. The visual acuity in our study remained the same in 90.0% and improved in 10.0% of patients after CK-SRS. The results of our series regarding LC and visual acuity are similar to those of a previous study on CK-RRS for ONSM by Marchetti et al. [27]. In this study, hypofractionated RRS with five fractions and a cumulative dose of 25 Gy was applied to 21 patients. They obtained a 100.0% LC rate, with 10.0% PR on post-radiosurgery MRIs with a mean follow-up time of 30.0 months. Similar to our study, there was no deterioration in visual function in any of these patients, but more patients (35.0%) achieved an improvement in their visual function with stable vision in 65.0% of patients. Nevertheless, both in our study and in the study cited, the follow-up was not long enough to rule out potential long-term damage to the optic nerves due to late toxicity of irradiation. Thus, further studies on this are needed.

With regards to side effects, our findings of mild headache (*n* = 1) and complaint of transient diplopia (*n* = 1) responding to 4 mg of dexamethasone and no other radiation-induced acute or late toxicities are consistent with those previously reported by Adeberg et al. [15], who reported only one patient who presented with mild optic neuropathy that recovered after administration of steroid therapy. The only patient with an infield tumor progress operated 70.9 months after RRS was a 30.9-year-old man with two prior surgeries and blindness prior to CK treatment. The patterns of failure analysis showed that this patient had the largest PTV volume and histopathologically expressed two atypical criteria (patternless growth and necrosis), which in the opinion of pathologists are not sufficient for a higher grade but their presence may be of increased risk for progression or recurrence.

The majority of studies on ONSM used adjuvant or primary FSRT with a cumulative dose of 50.4–54.0 Gy (28–30 fractions) [1,7,8,9,10,11]. For FSRT, the mean EQD2_2_ of the ipsilateral optic nerve and EQD2_3_ of the PTV would be 47.9–51.3 Gy and 48.4–51.8 Gy, respectively. In our series, the maximal EQD2_2_ dose to the ipsilateral optic nerve was 43.8 Gy (for patients with residual vision) and the mean EQD2_3_ of the PTV was 46.8 Gy. FSRT PTV doses were nominally slightly higher compared to the CK-SRS without consideration of acceleration and hypofractionation of radiosurgery. The series of patients who have received FSRT published since 2011 are shown in Table 5. In this context, we must point out that our aim was not to present all of the literature on the subject, but to provide an overview of the most recent publications for comparison with our data. All of them reported an LC rate of at least 98.0–100.0% at 5- and 10-years [1,15,30,31,32,33]. Compared to our data of 100% vision preservation and only mild side effects, FSRT studies reported slightly lower rates of vision preservation and higher rates of acute and long-term side effects. We summarized these in Table 5 in comparison to the radiosurgery results demonstrated in Table 4. Briefly, the most common acute adverse events were alopecia in up to 58%, headache in up to 32%, fatigue in up to 23%, nausea in up to 15%, dizziness in up to 12%, increased intracranial pressure in up to 10%, and eye problems (conjunctivitis, dry eye, and visual impairment) in up to 7% of patients. The main long-term side effects were ocular, including visual deterioration in up to 19%, hypopituitarism in up to 13%, dry eye in up to 12%, hyperlacrimation in up to 8%, retinopathy in up to 4–7%, ocular pain in up to 5%, scotoma in up to 3%, and obstructive hydrocephalus in up to 3% of patients [1,15,30,31,32,33].

The most common side effects due to radiosurgery, except for reversible conjunctiva edema in 13% and abnormal lacrimation in 10% of the patients, were in the low percentage range (dizziness in 5%, transient double vision with mild optic neuropathy in 4–5%, and transient mild headache in 3–4% of patients; Table 4) [18,25,26,27]. For RRS, it is important to respect the dose constraints to achieve low toxicity rates. Milano et al. [29] recently summarized 34 studies with a total of 1578 patients on dose constraints for the optic system.

In our cohort, 36% of patients (33.3% of lesions) underwent surgery before CK-SRS in a “hybrid” approach reflecting the need for an interdisciplinary “hybrid” approach for larger tumors in this region. On the same topic, a recent systemic report by Henaux et al. [11] showed worsening of visual acuity in 56% of patients who underwent surgery on the intraorbital optic nerve portion, arguing for a non-surgical approach. Thus, a “hybrid” approach should only be performed in specialized centers for selected cases with larger tumors or unclear diagnosis despite 68Ga-DOTATOC-PET imaging. Importantly, more than half of the patients in our cohort presented for surgery with already severely impaired vision; therefore, early diagnosis and intervention is the key to avoid visual deterioration in these patients in the first place.

### 4.1. Optic Nerve Movement and Dose Uncertainties

The steep dose gradients in RRS require precise knowledge of the location of the OAR and PTVs and a high level of confidence in the planned dose. Slight movement or dose deformation can therefore result in relevant changes in the OAR dose. Optic nerve movement was estimated from two different scans. The resulting displacements ranged from 0.78 to 11.77 mm and were in the order of those measured by Moodley et al., who found an average displacement of 2.10 ± 0.97 mm on the *x*-axis and 2.20 ± 1.61 mm on the *y*-axis during a 3 min MRI sequence with a range of 0.7–7.5 mm. The larger movement of the ipsilateral (2.99 mm) compared to the contralateral optic nerve (1.88 mm) is probably due to the more difficult demarcation between the tumor and nerve on the ipsilateral side. However, this is only an estimate from two snapshots. The effect of this displacement on the maximum dose of the optical pathway was rather small with a dose difference of 0.1 ± 0.4 Gy (range: −0.5–1.1) per fraction. Xiang et al. [37] studied the physiological movement of the optic chiasm and measured displacements of approximately 0.50 mm anteroposteriorly and superiorinferiorly and 0.75 mm laterally. When applied to radiosurgical treatment plans targeting a peri-chiasmatic lesion resulted in an average increase in D_0.03cc_ of 22% and in absolute maximum chiasm dose of 14%. To minimize the risk of radiation-induced optic neuropathy, they consider either a lower dose constraint or a margin for the chiasm. This may also compensate for additional uncertainties caused by the inhomogeneous tissue structure in the orbital region [37]. We investigated the impact of the dose calculation algorithm used for treatment optimization and found comparable dose differences of 0.3 ± 0.2 Gy (range: 0.1–0.7) per fraction for the optic nerve maximum dose. Doses calculated with the clinically used Ray-Tracing algorithm were always lower and therefore may underestimate the real dose to the optic pathway. However, the exact delineation of the ipsilateral nerve is difficult. Furthermore, the nerve also moves during the 30–60-min treatments and across fractions, so the maximum dose is likely to be somewhere between the calculated theoretical maxima. Despite these uncertainties, the visual results after SRS are good, indicating reasonable dose constraints.

### 4.2. Limitations

The main limitations of this study are the retrospective design and the limited number of patients because of the low incidence of ONSM. Our follow-up length was relatively short for benign tumors but longer than that in the previous studies of CK-RRS for ONSM. A very long-term follow-up would be desirable for a definitive evaluation of the technique. Nevertheless, it should be considered as a proof-of-concept study of CK-RRS for patients with ONSM to gain satisfactory local control and provide a low risk for treatment-related side effects. With the largest published lesion series of ONSM patients treated with RRS, our study provides valuable data for this patient group.

## 5. Conclusions

CK-RRS is technically feasible for the treatment of ONSM with excellent local control and stable or improved visual acuity at a medium follow-up. As an outpatient procedure, it combines good clinical results and vision preservation with only mild side effects. In particular, hypofractionated CK-RRS is an effective and safe alternative to surgery or FSRT. Further studies, preferably multicenter, are needed to determine the long-term outcomes of this treatment.

## Figures and Tables

**Table 1:** Summary of clinical characteristics for 27 lesions in 25 patients.

Characteristics	Number of Patients/Lesions (% of 25/27)
Gender	
Male	4 (16.0%)
Female	21 (84.0%)
Side	
Left	10 (37.0%)
Right	17 (63.0%)
Growth Pattern	
Circularly	25 (92.6%)
Peripherally	2 (7.4%)
Involvement of optic canal	
Orbital	11 (40.7%)
Canalicular	2 (7.4%)
Both	14 (51.9%)
Previous surgery	
Yes	9 (33.3%)
No	18 (66.7%)
Visual acuity (WHO category)	
0	10 (37.0%)
1	3 (11.1%)
2	3 (11.1%)
3	0 (0.0%)
4	4 (14.8%)
5	7 (25.9%)
Exophthalmos	
Yes	8 (29.6%)
No	19 (70.4%)
Restricted mobility	
Yes	8 (29.6%)
No	19 (70.4%)
Visual field restriction	
Yes	12 (44.5%)
No	5 (18.5%)
Blind/dark-bright	10 (37.0%)

**Table 2:** Comparison of the different cohorts in regard to treatment and outcome. Visual outcome categorized according to the World Health Organization (WHO) categories separated by previous surgery and Cyberknife (CK) fractionation (reported as median, range).

	Follow-Up Time (Month)	Visual Acuity (WHO) before CK	Visual Acuity (WHO) after CK	PTV (cm^2^)	Estimated Mobility of Ipsilateral Optic Nerve (mm)
Previous surgery					
yes (*n* = 9)	23.4 (6.4–78.7)	4 (1–5)	4 (0–5)	1.6 (0.7–14.1)	3.6 (1.7–11.8) *
no (*n* = 18)	30.5 (8.0–83.7)	0 (0–5)	0 (0–5)	0.8 (0.1–3.6)	2.5 (0.8–6.4)
Fraction scheme					
1 (*n* = 4)	49.1 (8.2–74.7)	5 (5–5)	5 (5–5)	2.8 (1.7–14.1)	3.7 (2.3–5.1) *
4–5 (*n* = 23)	25.8 (6.4–83.7)	1 (0–5)	0 (0–5)	0.9 (0.1–3.6)	3.0 (0.8–11.8)

*n* = number of patients; * *n*–2 because of missing data.

**Table 3:** Equivalent doses for 2 Gy fractions of the target volume and relevant organs at risk (*n* = 16, only WHO category < 5).

	EQD2 in Gy (*n* = 16, only WHO Category < 5)	
Organ	Mean	Median (Min–Max)
PTV		
D_min_	35.2	34.0 (30.5–46.8)
D_mean_	41.7	39.7 (36.1–53.0)
D_max_	49.6	48.0 (40.7–70.9)
Ipsilateral optic nerve *		
D_max_	43.8	43.1 (26.5–62.0)
D_mean_	20.8	18.6 (5.5–44.1)
Optic chiasm		
D_max_	3.9	1.4 (0.1–27.9)
Contralateral optic nerve **		
D_max_	1.8	1.3 (0.5–5.3)
Ipsilateral Retina/eye		
D_max_	15.7	5.3 (0.6–48.0)
Ipsilateral lens		
D_max_	0.5	0.4 (0.2–1.3)

EQD2 equivalent dose in 2 Gy per fraction; PTV planning target volume; D_max_/D_min_ near maximum and near minimum dose. * *n* = 15, because 1 could not be delimited. ** *n* = 14, because 2 patients were treated on both sides.

**Table 4:** Overview of all published radiosurgery studies of ONSM concerning local control and side effects.

First Author/Study Type	Year	Number of Patients	Radiation Device	Number of Fractions	Total Dose in Gy	Follow up in months	Local Control	Side Effects
Senger C./Retrospective (actual study)	2021	25 [27 lesions]	Cyberknife	1 4–5	14–15 (70% isodose) 20–25 (70–85% isodose)	37 (range: 6–84)	96% (11% remission, 85% stable, 4% progression)	mild headache 4%, transient diplopia 4%
Marchetti M. [27]/Prospective	2011	21	Cyberknife	5	25.0 (75%–85% isodose)	30 (range: 11–68)	100% (10% showed tumor shrinkage)	abnormal lacrimation 10% *, temporary diplopia with mild optic neuropathy 5% *, dizziness 5% *
Romanelli P. [18]/Retrospective	2011	5	Cyberknife	4	20.0 (70% isodose)	36–74	100%	n/a
Liu, D. [26]/Retrospective	2010	13 pONSM [17 sONSM; total: 30]	Gammaknife	1–2	13.3 (range 10.0–17.0)	56	93,3% at 5 years (66% regression 27% stable, 7% progression)	reversible conjunctival oedema 13% *, transient orbital pain 3% *, transient headache 3% *
Romanelli P. [25]/Retrospective	2007	3	Cyberknife	4	20.0 (80% isodose)	37	100% at 3 years	n/a

p/sONSM primary/secondary optic nerve sheath meningioma; n/a not available. * Percentage not indicated by study, self-calculated for facility comparison.

**Table 5:** Overview of recently (since 2011) published fractionated stereotactic radiotherapy studies for ONSM.

First Author/Study Type	Year	Number of Patients	Radiation Device	Total Dose in Gy	Follow-Up in Months	Local Control	Acute Side Effects	Long Term Side Effects
Kheir V. [31]/Retrospective	2019	16	n/a	50.4	31 (range: 2–156)	100% (79% * stable, 21% * reduced) (radiological reports of 88% * of pat.)	none	none
Ratnayake G. [30]/Retrospective	2019	26	Novalis TX (ExacTrac)	50.4 (range 50.4–54.0; 37.5 in 5–10%)	68 (range 20–134)	100% (96.1% stable, 3.8% mild reduction)	overall 53.8% of pat., fatigue 23.1%, headache 19.2%, alopecia 3.8%, dizziness 3.8%	dry eye 11.6%, retinopathy 4% *, multiple intracranial meningiomas 4% *
Hamilton S. N. [32]/Retrospective	2018	23 pONSM (18 sONSM; total: 41)	6 MV linear accelerator	50.0 and 50.4	45.6 visual assessment, 52.8 MRI	100% at 5 years for pONSM	headache 32%, nausea 15%, conjunctivitis 7%, dry eye 5%, eye discomfort 2%	hypopituitarism 13% of pONSM, retinopathy 7%, ocular pain 5%, cataract 2% (no differentiation of p/sONSM)
Paulsen F. [1]/Retrospective	2012	37 pONSM (76 sONSM; total: 109)	6 MV linear accelerator (Philips SL 25, Elekta Precise)	54.0 (range: 50.4–54.0)	30.2 clinical, 42.7 radio–graphic, 53.7 ophthalmologic	100% at 3 years and 98% at 5 years	alopecia 58% *, erythema 34% *, pain 28% *, vertigo 12% *, nausea 10% *, moderate increased cranial pressure 10% *	obstructive hydrocephalus 3% * with sONSM
Soldà F. [33]/Retrospective	2012	45	6 MV linear accelerator	50.0	30 (range: 12–156)	100% at 5 years	tiredness, small patches of transient alopecia	retinopathy 4%
Adeberg S. [15]/Prospective	2011	40	6 MV linear accelerator (Siemens)	54.0 (range: 25–66)	60 (range: 4–228)	100%	local alopecia (most pat.), fatigue 20% *, xerophthalmia 5% *, conjunctivitis 3% *	new headaches 3% *, hyperlacrimation 8% *, changed taste perception 3% *, scotoma and visual disorder 3% *

pat patients; p/sONSM primary/secondary optic nerve sheath meningioma; n/a not available. * Percentage not indicated by study, self-calculated for facility comparison.

## Data Availability

The datasets generated during and/or analyzed during the current study are not publicly available due to the protection of data privacy but are available from the corresponding author upon reasonable request as an anonymous set.
